# Changes in Gene Expression Profiling of Apoptotic Genes in Neuroblastoma Cell Lines upon Retinoic Acid Treatment

**DOI:** 10.1371/journal.pone.0062771

**Published:** 2013-05-01

**Authors:** Jon Celay, Idoia Blanco, Paula Lázcoz, Mirja Rotinen, Javier S. Castresana, Ignacio Encío

**Affiliations:** 1 Department of Health Sciences, Public University of Navarra, Pamplona, Spain; 2 Brain Tumor Biology Unit, University of Navarra School of Sciences, Pamplona, Spain; Johns Hopkins University, United States of America

## Abstract

To determine the effect of retinoic acid (RA) in neuroblastoma we treated RA sensitive neuroblastoma cell lines with 9-*cis* RA or ATRA for 9 days, or for 5 days followed by absence of RA for another 4 days. Both isomers induced apoptosis and reduced cell density as a result of cell differentiation and/or apoptosis. Flow cytometry revealed that 9-*cis* RA induced apoptosis more effectively than ATRA. The expression profile of apoptosis and survival pathways was cell line specific and depended on the isomer used.

## Introduction

During nervous system development several neuronal cells die due to apoptosis, thus regulating the appropriate number of neurons. The deregulation of this process and the subsequent inability of tumour cells to enter apoptotic pathways may lead to the appearance and progression of tumors, for which the restoration of a normal apoptotic rate represents an important antineoplastic therapy in tumor treatment. Retinoids exert essential effects during embryonic development and differentiation of several cell types [1], [2] and have been studied as human cancer therapeutics as they inhibit cell proliferation and induce cell differentiation and apoptosis in several cancer types including neuroblastoma [3], [4]. Different RA isoforms have been used for the treatment of neuroblastoma, either alone or in combination with chemotherapy or radiation, exerting their anti-tumor effects against neuroblastoma by the induction of apoptosis and/or differentiation [5]–[10]. *In vitro* 9-*cis* RA and ATRA isomers are the most common natural metabolites employed to induce apoptosis and/or differentiation of neuroblastoma cell lines [11]. Interestingly, the effect of RA has been reported to be dependent on the cell line treated, being some of them sensitive and others resistant to RA. For instance, it has been observed that SK-N-MC cells are sensitive to ATRA induced apoptosis after short periods of time (up to 72 h), while SK-N-FI cells are resistant to the same treatment [8]. Furthermore, treatment of SH-SY5Y cells induces their differentiation while suppression of the treatment favours their entrance in the apoptotic pathway [12]–[15]. In contrast, SK-N-AS cells do not show the morphological alterations typical of differentiated cells and only show a slight inhibition of cell growth after 8 days of exposure to RA [16], a reason for which this cell line is usually employed as a RA resistant control.

Apoptosis may be induced either by extrinsic or intrinsic pathways. The extrinsic or membrane receptor pathway is initiated by the interaction of several membrane receptors such as TNF Receptors, TRAIL Receptors, FAS or Death Receptors (DR) with their ligands. After binding, the transformed receptor induces both activator and effector caspases (caspases 8 and 10 and caspases 3, 6 and 7, respectively). The intrinsic or mitochondrial pathway is activated in response to different cell stimuli that lead to the release of cytochrome C from the mitochondria, the formation of the apoptosome complex and the activation of effector caspases [17], [18]. Besides the activation of the extrinsic pathway, the interaction of TNF with its receptor can also initiate a phosphorylation cascade that leads to NFKB1 transcription factor activation, which would in turn induce transcription of inhibitor of apoptosis proteins (IAPs) such as BIRC2, 3, 4, BCL2 or BCL2L1, therefore favouring cell survival [19], [20].

Here we report the effect on gene expression of apoptosis related genes during the processes of cell apoptosis and differentiation induced in neuroblastoma cell lines by treatment with either 9-*cis* RA or ATRA. Both by TUNEL and qPCR array, 9-*cis* RA revealed to be more efficient than ATRA inducing these processes. Furthermore, the qPCR array revealed that RA affects the expression of a different set of genes depending on the RA isomer used and the treated cell line.

## Materials and Methods

### Cell Culture and Cell Lines

IMR-32, SH-SY5Y, SK-N-DZ, SK-N-Be(2) and SK-N-AS neuroblastoma cell lines were obtained from ATCC (ATCC; Manassas, VA). The cell lines were chosen because their different RA sensitivity and genomic characteristics: IMR-32, SH-SY5Y, SK-N-DZ, SK-N-Be(2) cells are RA sensitive, while SK-N-AS cells were used as a negative control for RA treatment; SK-N-AS and SH-SY5Y cells harbour a single copy of MYCN while IMR-32, SK-N-DZ and SK-N-Be(2) cells show MYCN amplification at different extents. Additionally, one allele of CASP8 is hypermethylated in IMR-32, SK-N-DZ, SK-N-Be(2) and SH-SY5Ycells [21], [22]. Each cell line was cultured at 37°C in an atmosphere containing 5% CO_2_ and 96% relative humidity in DMEM Glutamax (Invitrogen, Carlsbad, CA) culture medium supplemented with previously decomplemented fetal bovine serum (Invitrogen) at a final concentration of 10%, and with non essential amino acids (Invitrogen) at a final concentration of 5%. Plasmocin (Invivogen, San Diego, CA) was used as antibiotic at a final concentration of 5 µg/ml. Cells were cultured in 75 cm^2^ flasks (Nunc, Roskilde, Denmark) and medium was changed every 48 h.

### RA Treatment

For retinoic acid treatment, cells were seeded in 75 cm^2^ flasks at 1.5 million cells/flask final concentration. After 24 h cells were washed and medium replaced by fresh medium including either the corresponding isomer of RA, 9-*cis* RA (Sigma, St. Louis, MO) or ATRA (Sigma) at a final concentration of 1 µM, or the vehicle (70% ethanol in the case of 9-*cis* RA, and DMSO in the case of ATRA) at a final concentration of 0.1%. Cells were treated for 9 days, or for 5 days followed by 4 days in which cells were maintained in absence of RA. Cell medium was changed every 48 h. At every change, apoptotic cells were recovered from old medium by centrifugation (5 min, 1,500 rpm) and re-added to the culture with the new medium.

### Apoptosis Detection

Apoptosis was determined by flow cytometry using the Apodirect Kit (BD Biosciences, San Diego, CA), which is based on the TUNEL technique under the conditions described by the manufacturer. Briefly, as neuroblastoma cells need at least 7 days to differentiate *in vitro* [23], cells were treated as described above either for 9 days or for 5 days followed by 4 days in absence of RA. Apoptosis was measured at days 1, 3, 5, 7 and 9. With this purpose cells were trypsinized, fixed in 1% paraformaldehide, stained with propidium iodide and FITC and analyzed in an EPICS-XL-4CLR flow cytometer (Beckman Coulter, Miami, Florida, USA). Cells were excited with an argon laser emitting at 488 nm and propidium iodide was detected using 620 nm band pass filter.

### RET and MYCN Expression Detection

To determine whether RA induced cell differentiation, RET and MYCN expression levels were measured by quantitative PCR (qPCR). Cells were treated as described above for 9 or 5 days. At days 1, 3, 5, 7 and 9 cells were trypsinized and collected by centrifugation. RNA was extracted from a maximum of 5 million cells per sample using the RNeasy extraction kit (Qiagen, Valencia, CA) according to the manufacturer’s instructions. After RNA extraction, 1 µg of total RNA was retrotranscribed using the SuperScript™ Reverse Transcriptase (Invitrogen) in the conditions described by the manufacturer.

RET, MYCN and GAPDH primers were designed using the “Beacon Designer 3.01″ software. Primer sequences were:

GAPDH-F: 5′GGAGTCCACTGGCGTCTTC3′

GAPDH-R: 5′ATCTTGAGGCTGTTGTCATACTTC3′

RET-F: 5′CATGGGCGACCTCATCTC3’

RET-R: 5′GAAATCCGAAATCTTCATCTTCC3′

MYCN-F: 5′ACCAGCGGCGGCGACCAC 3′

MYCN-R: 5′GTTCTTGGGACGCACAGTGATGG 3′

Each amplicon was cloned into a pGEM T vector to create pQRTGAPDH, pQRTRET and pQRTMYCN plasmids. These plasmids were transformed in JM109 High Efficiency Competent Cells (Promega, Madison, WI). The efficiency of each primer set was calculated by making quantitative PCRs using serial dilutions (2–0.002 ng of DNA) of each plasmid as template.

To study the expression of the selected genes in the cell lines treated with RA we performed quantitative PCRs. The expression of RET, MYCN and GAPDH was measured using the Brilliant SYBR Green QPCR Master Mix (Stratagene, La Jolla, CA). Briefly, the master mix was mixed with the primers at a final concentration of 5 µM, and 14.5 µl of the mix were added to each well, adding 2 ng of cDNA diluted in 10.5 µl of water to each well. The qPCRs were performed in a chromo 4 thermal cycler (MJ Research, Bio-Rad, Hercules, CA) in the following conditions: one cycle of 10′ at 95°C, to activate the HotStart DNA polymerase, followed by 40 cycles of denaturation at 95°C for 30 s and annealing and extension at 60°C for 30 s. SYBR Green fluorescence was measured at every cycle.

### qPCR Arrays

Cells were treated with RA as previously described, and trypsinized after 5 days of treatment, collected by centrifugation, and RNA extracted as described above. After RNA extraction, 1 µg of total RNA was retrotranscribed to cDNA using the RT^2^ First Strand Kit (Superarray, Hilden, Germany) as described by the manufacturer. Real time PCR was performed using the RT^2^ Profiler PCR array system focused on the expression of cell survival and apoptotic genes (Ref.: PAHS-012, Superarray, Hilden, Germany). The RT^2^ qPCR Master Mix (Superarray) was prepared in the conditions described by the manufacturer. Briefly, a master mix containing 1 µg of cDNA was prepared, and 25 µl of the mix were added to each well of the PCR array. The qPCR was performed in a Chromo 4 thermal cycler (MJ Research, Bio-Rad, Hercules, CA) in the following conditions: one cycle of 10 min at 95°C, to activate the HotStart DNA polymerase, followed by 40 cycles of denaturation at 95°C for 15 s, annealing at 55°C for 30 s, and extension at 72°C for 30 s. SYBR Green fluorescence was measured in every cycle.

### Statistical Analysis

We performed at least four duplicated independent experiments for the TUNEL assay and three independent experiments for the qPCRs, which were analyzed using the ΔΔC(t) method. Distribution of the samples was analyzed by using the Shapiro Wilks and Kolmogorof-Smirnov tests and for statistical significance of the gene expression variation the student t test taking p<0.05 as the criterion for significance. Data were analyzed using the SPSS 15.0 program for Windows. Results are expressed as mean ± SEM.

## Results

### 9-*cis* RA is a More Efficient Apoptosis Inducer than ATRA

**Figure 1:**
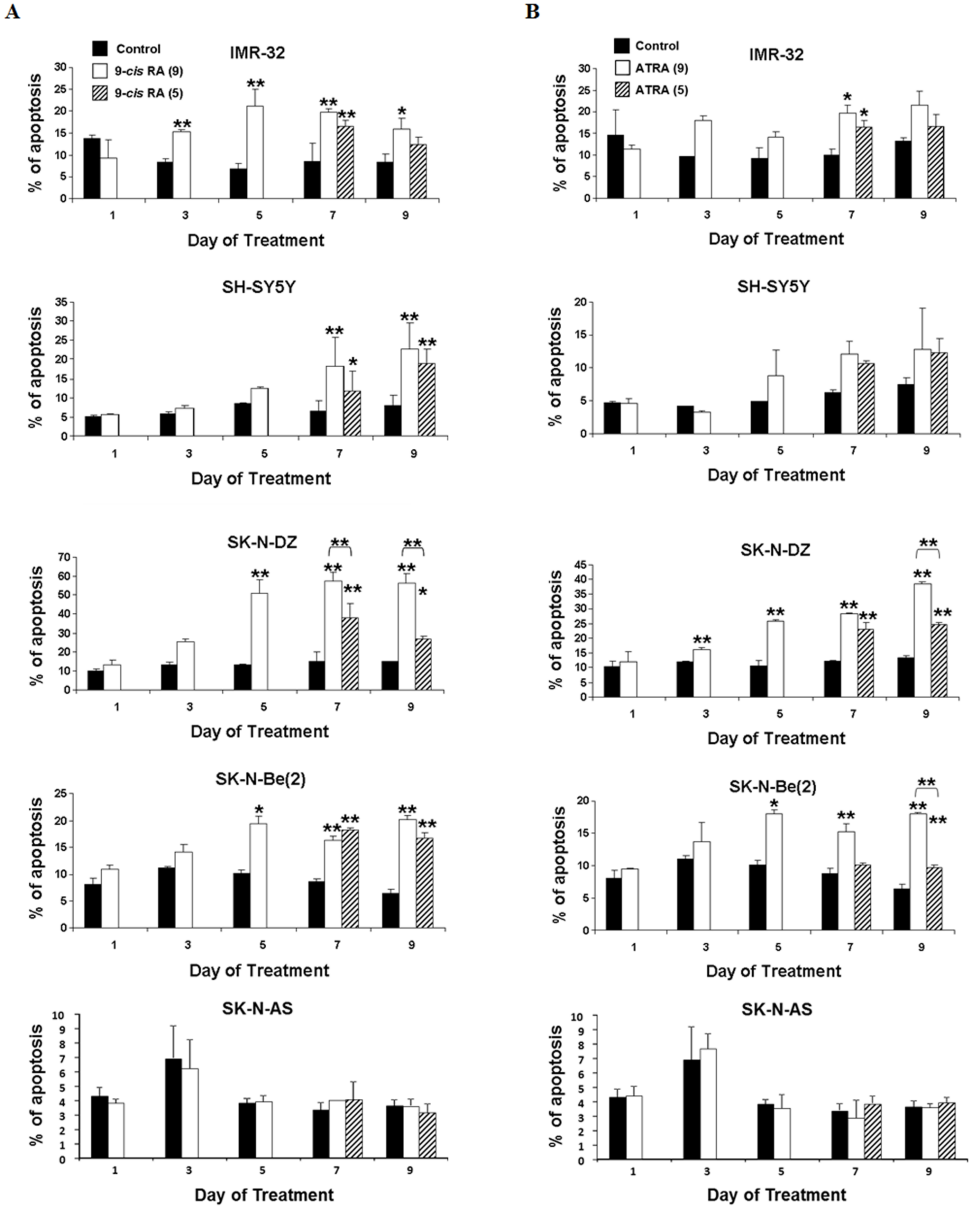
Time course analysis of induction of apoptosis in neuroblastoma cell cultures by Retinoic Acid. Percentage of dead cells in IMR-32, SH-SY5Y, SK-N-DZ, SK-N-Be(2) and SK-N-AS cell cultures either untreated (control cells) or treated with (A) 9-*cis* RA or (B) ATRA for 9 consecutive days (RA(9)) or for 5 days followed by 4 days in the absence of RA (RA(5)) was determined by TUNEL after 1, 3, 5, 7 or 9 days of treatment. Data are represented as mean ± SEM (n = 3). Asterisks represent significant differences to the control (*, p<0.05; **, p<0.01).

To compare the ability of different RA isomers to induce apoptosis of neuroblastoma cells, we measured the apoptotic status of the cells in IMR-32, SH-SY5Y, SK-N-DZ and SK-N-Be(2) cell cultures treated with either 1 µM 9*-cis* or ATRA for 9 days or for 5 days as described above. The RA resistant SK-N-AS cell line was used as a negative control. As shown in Figure 1A, treatment with 9-*cis* RA induced a significant increase in the percentage of apoptotic cells in all the cultures but SK-N-AS. The increase in the apoptotic level was observed from the 3^rd^ day in IMR-32 cell cultures, from the 5^th^ day in SK-N-DZ and SK-N-Be(2) cell cultures and from the 7^th^ day in SH-SY5Y cell cultures. Moreover, while the percentage of apoptotic cells sustained over treatment, suppression of 9-*cis* RA on the 5^th^ day reduced the percentage of apoptotic cells in IMR-32 and SK-N-DZ cultures but not in SH-SY5Y and SK-N-Be(2) cultures, thus indicating that the presence of RA is necessary for the maintenance of apoptosis in IMR-32 and SK-N-DZ cells. In the case of treatment with ATRA, while treatment did not induce a significant increase of apoptosis in SH-SY5Y cell cultures, induction of apoptosis started on the 3^rd^ day in SK-N-DZ cell cultures, on the 5^th^ day in SK-N-Be(2) cell cultures and on the 7^th^ day in IMR-32 cell cultures (Figure 1B). The percentage of apoptotic cells persisted after suppression of the treatment in the four cell lines.

**Figure 2:**
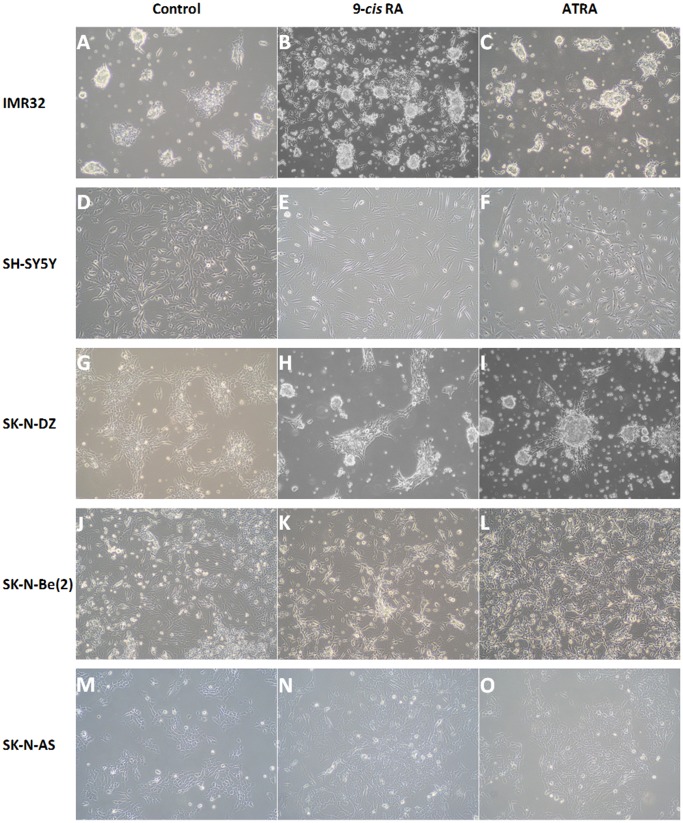
Morphological alterations induced in neuroblastoma cell lines treated with retinoic acid. Control cells (A, D, G, J and M) and cells treated with 9-*cis* RA (B, E, H, K and N) or ATRA (C, F, I, L and O) are shown. The images shown were taken at different time points: A: Day 1; B: Day 9; C: Day 5; D: Day 5; E: Day 3; F: Day 9; G: Day 1; H: Day 3; I: Day 5; J: Day 9; K: Day 9; L: Day 9; M: Day 1; N: Day 7; O: Day 5; All images were taken at 100X.

Previous works have described that both 9-*cis* and all *trans* retinoic acid isomers induce differentiation in neuroblastoma cell lines [24]. To determine whether RA effectively induced differentiation apart from apoptosis in our experimental system, we analyzed the morphological alterations of the cells observed during the treatment. As shown in Figure 2, while no morphological changes were observed in the RA resistant SK-N-AS cell cultures, exposure to RA of IMR-32, SH-SY5Y, SK-N-DZ and SK-N-Be(2) cells induced an augmentation in the number and length of the neuritic extensions, which can even connect with the extensions of neighbour cells. These alterations in cell morphology are typical of cells undergoing differentiation and were more evident in the cells treated with 9-*cis* RA than in those treated with ATRA. Furthermore, as a result of the treatment total cell number in the cultures decreased, which correlates with both cell differentiation and the apoptosis described above.

**Figure 3:**
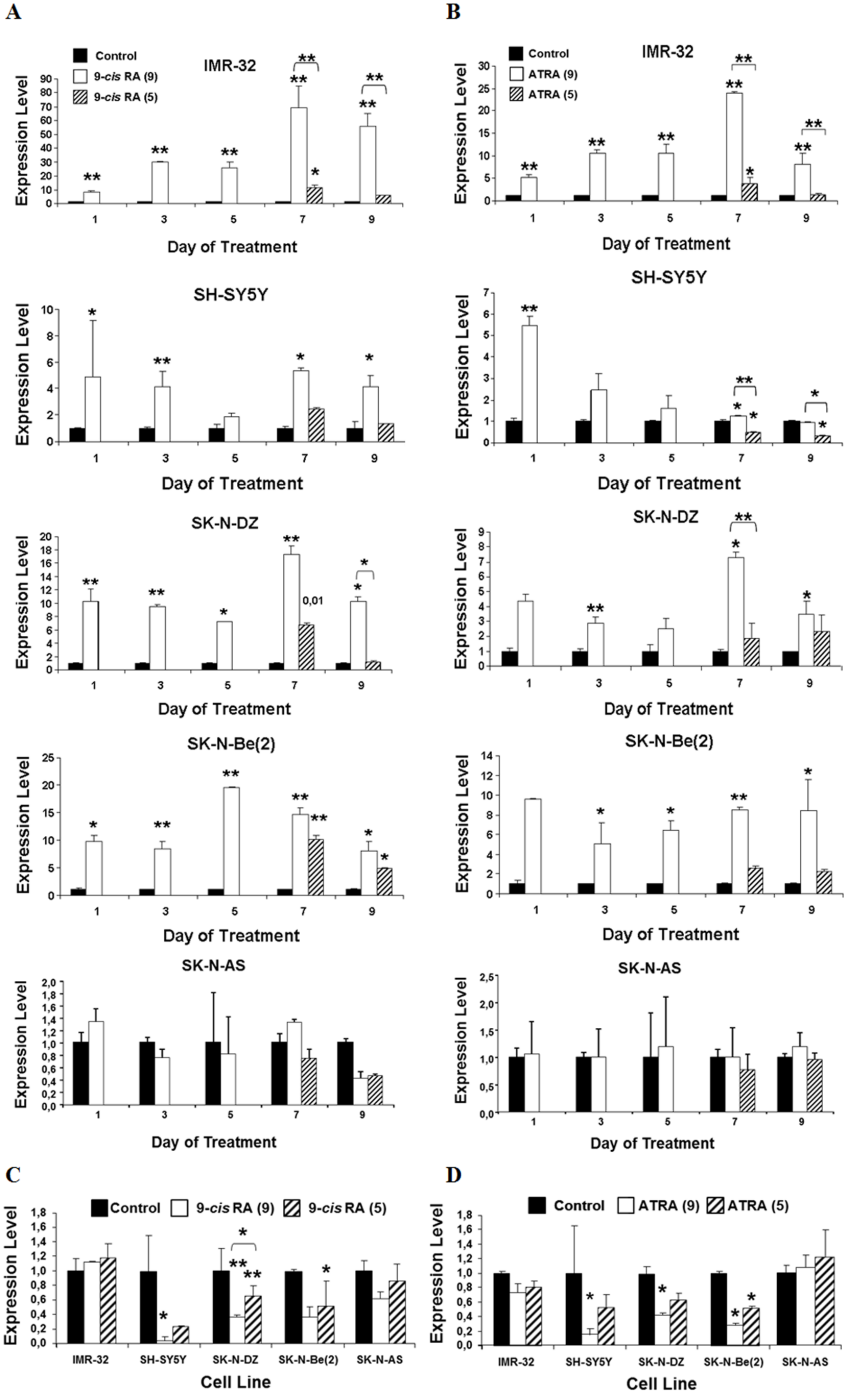
Time course analysis of the differentiation process induced in neuroblastoma cell cultures by Retinoic Acid. Expression levels of the oncogenes RET and MYCN were measured in IMR-32, SH-SY5Y, SK-N-DZ, SK-N-Be(2) and SK-N-AS cell cultures either untreated (control cells) or treated with 9*-cis* RA or ATRA for 9 consecutive days (RA(9)), or treated for 5 days followed by 4 days in the absence of RA (RA(5)). Expression levels were measured by qPCR as described in Materials and Methods and are shown relative to GAPDH. RET levels in cells either untreated (control) or treated with (A) 9-*cis* RA or (B) ATRA were determined at days 1, 3, 5, 7 and 9. MYCN levels in cells either untreated (control) or treated with (C) 9*-cis* RA or (D) ATRA were measured at day 9. Data are represented as mean ± SEM (n = 3). Asterisks represent significant differences to the control (*, p<0.05; **, p<0.01).

To confirm differentiation of the RA sensitive cell lines, the variation on the expression levels of the oncogenes RET [25]–[27] and MYCN [28], relative to GAPDH, were determined by qPCR by the ΔΔCt method [29]. Figure 3A and B show that both 9-*cis* RA and ATRA led to a significant increase in RET expression in every RA sensitive cell line, thus indicating the capability of RA to induce cell differentiation. Nevertheless, reached expression level was dependent on the treated cell line and the isomer used. Furthermore, we observed that the presence of RA is essential to maintain RET expression level, as the suppression of the treatment on the 5^th^ day led to a decrease in the expression of RET, reducing it to the same level than in control cells.

MYCN expression levels (Figure 3C and D) were measured the 9^th^ day of treatment, and both 9-*cis* RA and ATRA inhibited its expression in SH-SY5Y, SK-N-DZ and SK-N-Be(2) cells, but not in the RA resistant SK-N-AS cell cultures neither in the RA sensitive IMR-32 cells. Since the IMR-32 cell line harbours 2 different phenotypes, one of undifferentiated cells and the other of proliferative cells that we have not separated, this might be and explanation for the lack of MYCN down-regulation by RA in this cell line. Additionally, suppression of either 9-*cis* RA or ATRA on day 5 in SH-SY5Y, SK-N-DZ and SK-N-Be(2) cells lead to an increase in MYCN expression on day 9, that shows up at different extent on each cell line, as compared with those cells where treatment was maintained, indicating that the presence of RA is necessary to inhibit MYCN expression. These results suggest that the differences observed in the modification of MYCN expression among cell lines might in part be due to the different MYCN copy number that possesses each one and reflect the different capability of RA to induce cell differentiation depending on the cell type.

All together these results show that RA is able to induce apoptosis and differentiation in neuroblastoma cell cultures. Moreover, since higher percentages of apoptotic cells were detected in 9-*cis* RA versus ATRA treated cell cultures (p<0.05 at day 5 in SK-N-DZ and IMR32 cells; p<0.05 at day 7 and day 9 in SHSY5Y and SK-N-DZ cells treated continuously; p<0.05 at day 7 and day 9 in SHSY5Y, SK-N-DZ and SK-N-Be(2) cells treated until day 5), results also suggest that 9*-cis* RA is a more efficient apoptosis inducer than ATRA in neuroblastoma cells.

### The Treatment of Neuroblastoma Cell Lines with 9-*cis* RA Alters the Expression of a Heterogeneous Group of Genes

Since in most cases the level of apoptosis was significantly increased after 5 days of treatment with RA, we next decided to analyze the expression level of apoptosis related genes at that moment. Thus, we treated the different cell lines with 9-*cis* RA as previously described and, after treatment, we analyzed by qPCR (RT^2^ Profiler PCR array PAHS-012, Superarray) the expression level of 84 genes related to apoptotic and survival pathways. Obtained results are shown in Table 1. Only those genes whose expression augmented more than 2-fold or those whose expression was reduced to less than their half are displayed. Results show that the treatment altered the expression level of 20 genes in IMR-32 cells, where expression of 16 genes (BCL2L11, BIRC3, BIRC8, CARD6, CASP1, CASP5, CASP7, CASP10, CD40LG, CD70, FASLG, LTA, LTBR, TNFRSF10A, TNFRSF11B and TNFRSF21) was increased and expression of 4 genes (APAF1, BCL10, CASP6 and HRK) was inhibited. Thirteen genes were modified in SH-SY5Y cells, where 12 genes (BIK, BIRC3, CARD6, CASP1, CASP4, CD70, PYCARD, RIPK2, TNFRSF9, TNFRSF10A, TNFRSF10B and TNFSF10) were overexpressed and one gene (CD40) was downregulated. Thirteen genes were modified in SK-N-DZ cells, 11 of which (BCL2A1, BIRC3, CASP1, CASP5, CASP8, CASP10. CASP14, NOL3, TNFRSF9, TNFRSF11B and TNFSF10) increased their expression, while 2 (HRK and TNF) decreased it. Finally, expression of 9 genes was altered in the SK-N-Be(2) cells, 6 of which (BIK, LTBR, PYCARD, TNFRSF1A, TNFRSF10B, TNFRSF25) were overexpressed and 3 of which (BCL2L10, CD27 and TP73) were downregulated. Although the response of the 4 cell lines to the treatment with 9-*cis* RA was similar, i. e. induction of apoptosis, these results show that apoptosis related genes respond to 9-*cis* RA treatment in a very heterogeneous and cell line dependent manner.

**Table 1:** Genes of the apoptotic pathway overexpressed at least two times, or downregulated by 50%, after treatment of neuroblastoma cell lines with 9*-cis* RA.

Upregulated Genes	IMR32	SH-SY5Y	SK-N-DZ	SK-N-Be(2)
BCL2A1	–	–	2,077*	–
BIK	–	2,93**	–	3,105*
BIM	3,202*	–	–	–
CARD6	5,546**	5,633*	–	–
CASP1	2,704*	3,567**	3,251*	–
CASP10	2,922*	–	3,945*	–
CASP14	–	–	2,151**	–
CASP4	–	1,935*	–	–
CASP5	7,708*	–	4,193*	–
CASP7	2,62*	–	–	–
CASP8	–	–	4,889*	–
CD137	–	–	10,693*	–
CD40LG	7,24*	–	–	–
CD70	2,061*	5,818**	–	–
cIAP2	2,15**	5,766**	4,565*	–
DR3	–	–	–	4,492*
DR6	2,321**	–	–	–
FASLG	3,239*	–	–	–
ILP2	5,015*	–	–	–
NOL3	–	–	2,056*	–
PYCARD	–	2,873*	–	2,23*
RIPK2	–	3,226*	–	–
TNF-RI	–	–	–	4,854*
TNF-RIII	3,102*	–	–	5,469**
TNFRSF11B	2,414*	–	5,106*	–
TNFRSF9	–	5,164*	–	–
TNFβ	3,634**	–	–	–
TRAIL	–	26,057**	5,714**	–
TRAIL-RI	4,147*	2,731*	–	–
TRAIL-RII	–	2,756**	–	3,199*
Downregulated genes	IMR32	SH-SY5Y	SK-N-DZ	SK-N-Be(2)
APAF1	0,466*	–	–	–
BCL10	0,424**	–	–	–
BCL2L10	–	–	–	0,408*
CASP6	0,508*	–	–	–
CD27	–	–	–	0,455*
CD40	–	0,51*	–	–
HRK	0,48*	–	0,135**	–
TNF	–	–	0,346*	–
TP73	–	–	–	0,394*

Genes significantly upregulated or downregulated more than 2-fold are represented. Asterisks indicate significant differences among control and treated cells (*, p<0.05; **, p<0.01). Lines (–) indicate that these genes were neither overexpressed at least two times, nor downregulated by 50% after the treatment.

### ATRA Alters Both Apoptotic and Survival Pathways in Neuroblastoma Cell Lines

To study the effect of ATRA on the expression level of apoptosis related genes in neuroblastoma cell lines, we treated IMR-32, SH-SY5Y, SK-N-DZ and SK-N-Be(2) cell cultures as described above for 9-*cis* RA. After treatment gene expression levels were analyzed by qPCR. Obtained results are shown in Table 2. As shown, 7 genes were modified in IMR-32 cells, where 2 genes were upregulated (CIDEA and TNFRSF9) and 5 genes (APAF1, CD30L, TNFRSF1A, TNFRSF25 and TP53) downregulated. Eight genes were altered in SH-SY5Y cells; in this case the expression of 6 genes was increased (BIRC3, CASP1, CD70. CIDEA, TNFRSF10B and TNFSF10) and 2 genes were downregulated (APAF1 and BFAR). Expression of 8 genes was modified in SK-N-DZ cells, 7 of which were upregulated (CASP1, CASP5, CASP8, CASP10, CD40LG, TNFRSF11B and TNFSF10) and one gene was downregulated (HRK). Finally, 5 genes were modified in SK-N-Be(2) cells, 4 of which were upregulated (CASP3, CASP10, LTBR and TNFRSF25) and one gene downregulated (TP73). As described above for 9-*cis* RA, these results suggest that the effect of ATRA is specific of the cell line treated, since expression of various genes is differently altered in each particular cell line. Moreover, final response to the treatment with ATRA also seems to be dependent on the cell line treated, as ATRA did not induce a significant apoptotic increase in SH-SY5Y cells, while it did it in the rest of the lines: in IMR-32 cells ATRA induced a significant increase of apoptosis, but not comparable to the one observed in SK-N-DZ and SK-N-Be(2) cells, in which apoptosis levels increased among the 3^rd^ and 5^th^ day of treatment and sustained until the end of the treatment (Figure 1).

**Table 2:** Genes of the apoptotic pathway overexpressed at least two times, or downregulated by 50%, after treatment of neuroblastoma cell lines with ATRA.

Upregulated genes	IMR32	SH-SY5Y	SK-N-DZ	SK-N-Be(2)
CASP1	–	2,024**	3,464*	–
CASP10	–	–	3,905*	2,609*
CASP3	–	–	–	1,922*
CASP5	–	–	2,202*	–
CASP8	–	–	3,700*	–
CD137	3,523*	–	–	–
CD40LG	–	–	3,302*	–
CD70	–	5,257**	–	–
cIAP2	–	4,281**	–	–
CIDEA	1,938*	2,007*	–	–
DR3	–	–	–	2,432*
TNF-RIII	–	–	–	1,951*
TNFRSF11B	–	–	6,467*	–
TRAIL	–	2,952*	5,816**	–
TRAIL-RII	–	2,003*	–	–
Downregulated genes	IMR32	SH-SY5Y	SK-N-DZ	SK-N-Be(2)
APAF1	0,593*	0,53*	–	–
BFAR	–	0,572*	–	–
CD30L	0,238*	–	–	–
DR3	0,507*	–	–	–
HRK	–	–	0,126*	–
TNF-RI	0,288*	–	–	–
TP53	0,643**	–	–	–
TP73	–	–	–	0,341*

Genes significantly upregulated or downregulated more than 2-fold are represented. Asterisks indicate significant differences among control and treated cells (*, p<0.05; **, p<0.01). Lines (–) indicate that these genes were neither overexpressed at least two times, nor downregulated by 50% after the treatment.

## Discussion

In this work we have analyzed the effect of the treatment of neuroblastoma cell lines with 9-*cis* RA or ATRA in cell apoptosis and differentiation. The overexpression of RET and the inhibition of MYCN oncogenes as markers of cell differentiation [25]–[27] [28] and the effect of RA as an inducer of cell differentiation in neuroblastoma cell cultures [24] have been widely described. Accordingly, we have found that despite some specific differences in the response to 9-*cis* and ATRA among cell lines, both RA isomers induce the morphological alterations typical of cell differentiation and increase RET expression in every one of the RA sensitive tested cell lines (Figures 2 and 3), while MYCN expression was inhibited in three of the four cell lines (Figure 3). Additionally, we have also found that both RA isomers are able to increase the percentage of apoptotic cells in the cultures. However, the apoptotic response of the cells was quite heterogeneous. Thus, apoptosis induction by 9-*cis* RA showed up rapidly in IMR-32, SK-N-DZ and SK-N-Be(2) cells, where it began between the 3^rd^ and the 5^th^ day of treatment, while SH-SY5Y cells were more resistant (Figure 1A). Interestingly, other groups have reported induction of apoptosis by 9-*cis* RA in SH-SY5Y cells only after suppression of the treatment [12], [14]. In our study we also observed some increase in the percentage of apoptotic cells when the treatment was suppressed, although this increase was not comparable to the one observed when cells were treated continuously for a long length time. However, apoptosis level kept high in SK-N-Be(2) cells and decreased in IMR-32 and SK-N-DZ cell cultures when treatment was suppressed. These results suggest either that SH-SY5Y and SK-N-Be(2) cells are more sensitive to 9-*cis* RA than SK-N-DZ and IMR-32 cells, or that although apoptotic pathways are activated in the four cell lines, 9-*cis* RA would also induce the activation of survival pathways in SK-N-DZ and IMR-32 cells. On the other hand, when cells were treated with ATRA induction of apoptosis began after 3 and 5 days of treatment in SK-N-DZ and SK-N-Be(2) cell cultures, and only after 7 days in IMR-32 cell cultures (Figure 1B). However, ATRA treatment did not induce a significant increase of apoptosis in SH-SY5Y cell cultures. Furthermore, the percentage of apoptotic cells in the cultures maintained steady after the suppression of the treatment in the four cell lines. These results may indicate that SK-N-DZ and SK-N-Be(2) cell lines respond equally to both isomers and suggest that the same apoptotic pathways are activated in these lines after the treatment with any of the RA isomers. Results also suggest that ATRA is a less efficient apoptosis inductor than 9-*cis* RA in IMR-32 and SH-SY5Y cells.

**Figure 4:**
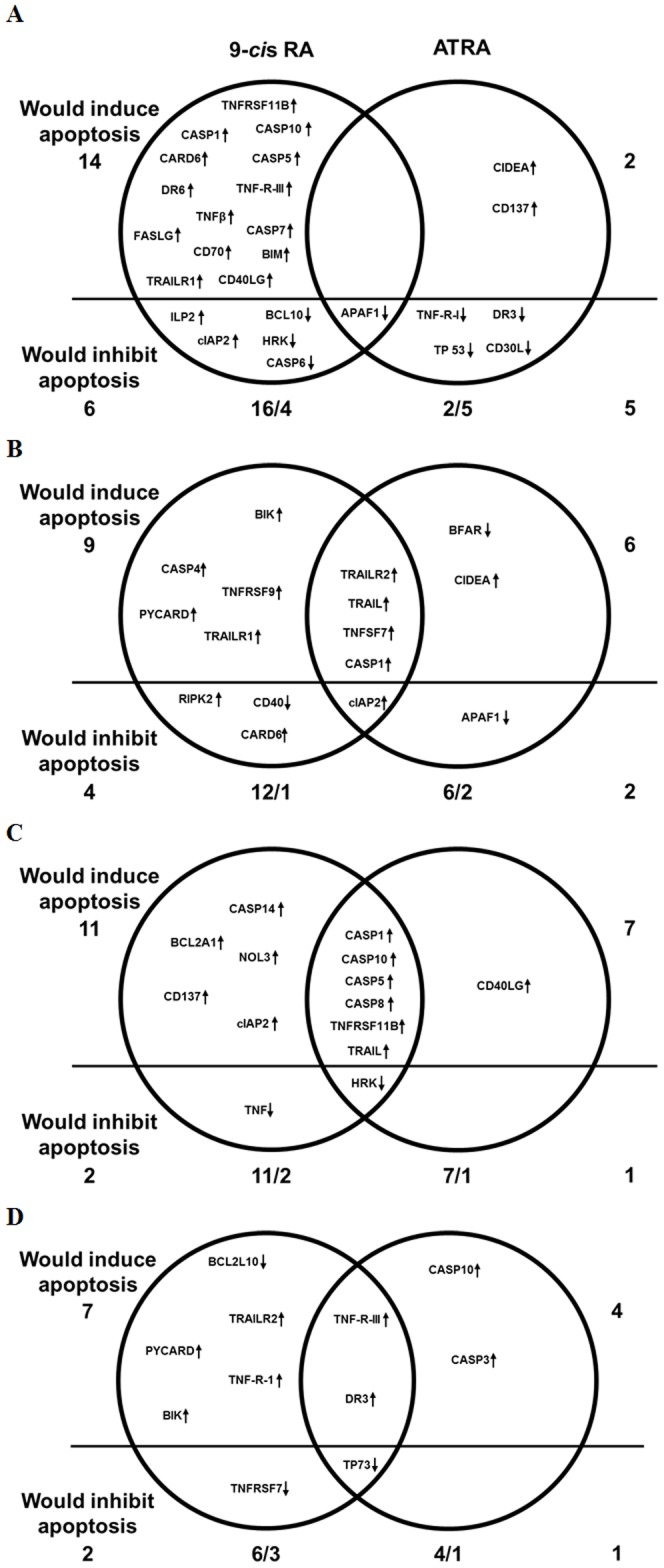
Edwards-Venn diagrams of genes of the apoptotic pathway whose expression was altered in neuroblastoma cell lines after RA treatment. Genes overexpresed at least 2 times (up arrows) or downregulated by 50% (down arrows) after 5 days of treatment either with 9-*cis* RA or ATRA. (A) IMR-32, (B) SH-SY5Y, (C) SK-N-DZ and (D) SK-N-Be(2). The line represents the division among the modified genes that would induce and inhibit apoptosis.

Analysis of the effect of RA on the expression profile of genes related to apoptosis and cell survival showed that modified genes had either proapoptotic or antiapoptotic actions. According to the modification (up or down-regulation) and function of each gene (pro or antiapoptotic) we divided them into two groups: those whose modification would induce apoptosis and those that would inhibit apoptosis. The first group was formed by proapoptotic genes whose expression was increased and by antiapoptotic genes whose expression was decreased after the treatment, while the second group was formed by antiapoptotic genes whose expression was increased and by proapoptotic genes whose expression was decreased after the treatment. According to this division we analyzed the relationship among the apoptosis level observed upon RA treatment (Figure 1) and the pro and antiapoptotic genes modified at each cell line by either of the RA isomers (Figure 4).

**Figure 5:**
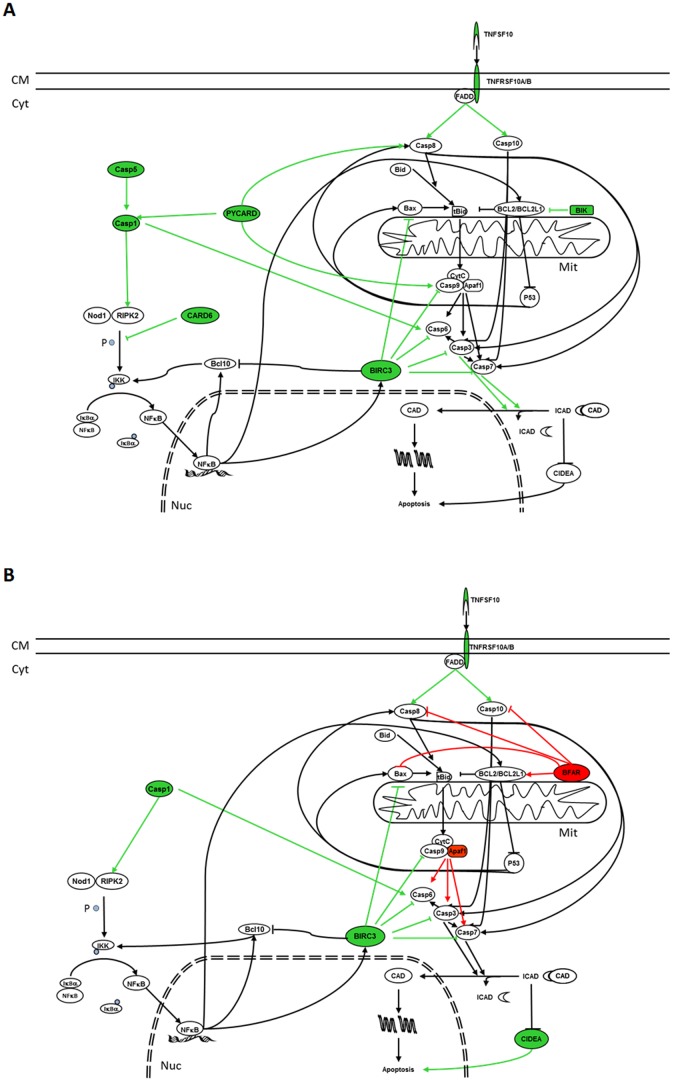
Effect of RA treatment over apoptotic and cell survival pathways in SH-SY5Y RA sensitive cell line. In green colour are represented those genes whose expression has been augmented as a result of 9-*cis* RA (A) or ATRA (B) treatment, while in red are shown those whose expression has been decreased. Green arrows indicate the pathways that will be activated while red arrows indicate the pathways that will be inhibited as a consequence of the modifications of the genes that take part in these pathways. CM: Cell Membrane; Cyt: Cytoplasm; Mit: Mitochondrion; Nuc: Nucleus; P: Phosphorous.

**Figure 6:**
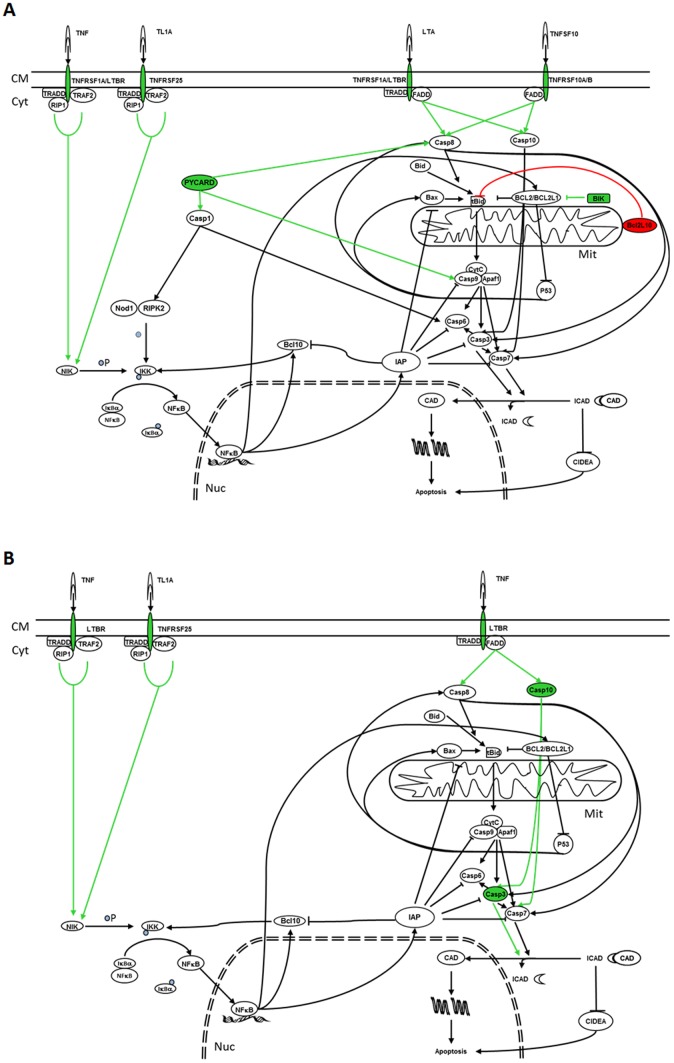
Effect of RA treatment over apoptotic and cell survival pathways in SK-N-Be(2) RA sensitive cell line. In green colour are represented those genes whose expression has been augmented as a result of 9-*cis* RA (A) or ATRA (B) treatment, while in red are shown those whose expression has been decreased. Green arrows indicate the pathways that will be activated while red arrows indicate the pathways that will be inhibited as a consequence of the modifications of the genes that take part in these pathways. CM: Cell Membrane; Cyt: Cytoplasm; Mit: Mitochondrion; Nuc: Nucleus; P: Phosphorous.

**Figure 7:**
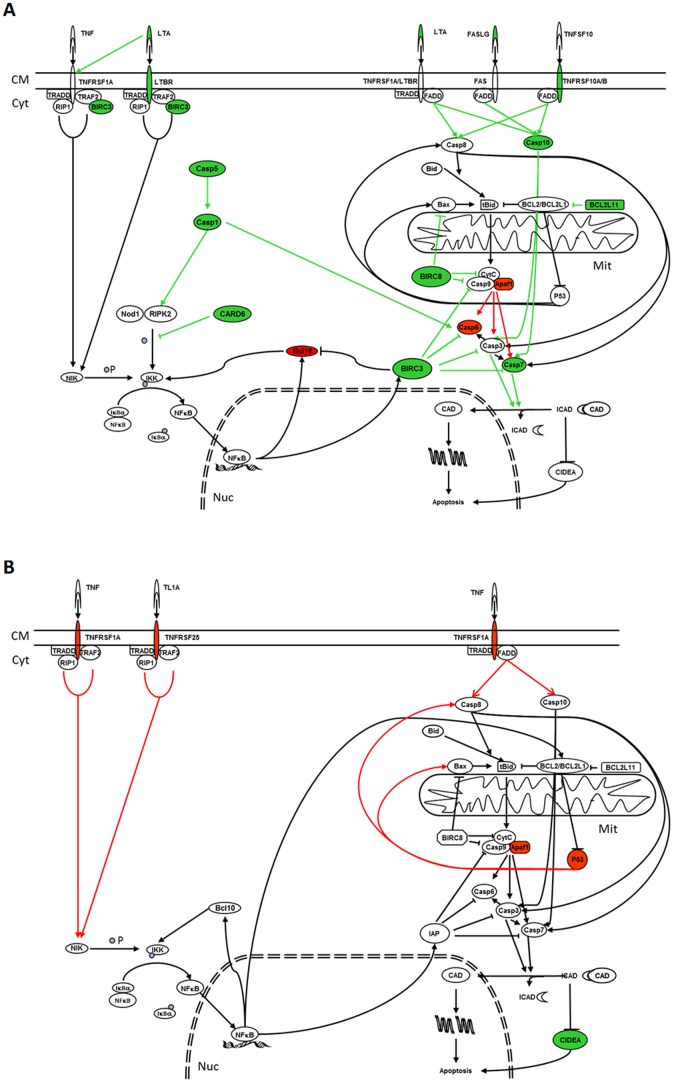
Effect of RA treatment over apoptotic and cell survival pathways in IMR-32 RA sensitive cell line. In green colour are represented those genes whose expression has been augmented as a result of 9-*cis* RA (A) or ATRA (B) treatment, while in red are shown those whose expression has been decreased. Green arrows indicate the pathways that will be activated while red arrows indicate the pathways that will be inhibited as a consequence of the modifications of the genes that take part in these pathways. CM: Cell Membrane; Cyt: Cytoplasm; Mit: Mitochondrion; Nuc: Nucleus; P: Phosphorous.

Analysis suggests that treatment with either 9-*cis* RA or ATRA induces apoptosis by the activation of the extrinsic and p53 pathways in SH-SY5Y and SK-N-Be(2) cells (Figures 5 and 6). However, only 9-*cis* RA would be able to activate these pathways in IMR-32 cells (Figure 7). Activation of the extrinsic pathway is supported by the fact that after treatment expression of TNF10 and its receptors TNFRSF10A or TNFRSF10B in SH-SY5Y cells, expression of LTBR, TNFRSF1A, TNFRSF10B or TNFRSF25 in SK-N-Be(2) cells and expression of TNFRSF10A, LTA and FASLG in IMR-32 are augmented. Furthermore, the elevation of the levels of the membrane receptors and their ligands in these cell lines is followed by the overexpression of CASP1, CASP3, CASP5, CASP7, CASP10 or PYCARD, which will favour the entrance of the cells into the apoptotic process. In SH-SY5Y cells treated with ATRA, downregulation of the caspase inhibitor BFAR will also contribute to the apoptotic process. Activation of the p53 pathway is supported by induction of BIK expression in SH-SY5Y and SK-N-Be(2) cells and of BCL2L11 expression in IMR-32 cells, since BIK and BCL2L11 are BCL2 interacting factors that block its antiapoptotic activity hence leading to the activation of p53 dependent genes [30]. However, activation of the apoptotic intrinsic pathway is less probable since treatment of IMR-32 and SH-SY5Y cells with 9-*cis* RA leads to an increase of BIRC3 and BIRC8 (an inhibitor of APAF1, BAX and Caspase 9 [31], [32]) and to a decrease of APAF1 and BCL10 (an activator of Caspase 9 [33]), thus suggesting a lesser formation of the apoptosome and a blockade of the pathway.

It has to be noticed that the IMR-32 cell line consists of 2 phenotypes: one phenotype is formed by a pool of undifferentiated cells, and the other by proliferative cells able to become adherent epithelial like cells [34]. Taking into account that in this work we have not separated the two phenotypes and that during the treatment we observed by microscopy that the undifferentiated cells entered apoptosis and the proliferative cells differentiated to adherent cells (Figures 2A, B and C), the analysis of the modifications on the expression profile of genes related to apoptosis and cell survival suggests that the treatment of IMR-32 with 9-*cis* RA would favour cell survival and differentiation in the proliferative cells through the activation of signalling pathways such as those regulated by NFKB1, p38, MAPK and/or JNK. The activation of these pathways in the undifferentiated cells seems less probable. Several facts support this idea. First, the expression level of CARD6 is increased as a consequence of the treatment, and it has been described that the interaction of CARD6 with NOD1 and RIPK2 suppresses the activation of NFKB1 induced by these two proteins and so its survival effect [35]. Second, BCL10 levels, a protein that regulates the NFKB1 signalling pathway [36], [37], decreased as a consequence of the treatment. Third, levels of CD40LG increased as a consequence of the treatment, and since it has been shown that CD40 activation causes TRAF2 degradation in B cells [38] this result suggests that treatment leads to a decrease in TRAF2 activity and so to a blockade in the NFKB1 and MAPK signalling pathways and the inhibition of their cell survival effects; the increase in the expression of BIRC3, an antiapoptotic protein capable of both binding TRAF2 and inhibit caspase activities [33], [39] could be the consequence of a compensatory mechanism that would try to maintain the function of these pathways; anyway, it has to be taken into account that the capability of BIRC3 to inhibit apoptosis is very weak and that the main reason of its binding to TRAF2 seems to be to mediate or modulate receptor signalling [31]. Fourth, caspase 1 levels increased as a consequence of the treatment but as previously commented, the activating effect of caspase 1 over RIPK2 [32] is probably counteracted by the elevation of CARD6. Overexpression of CARD6 and BIRC3 was also observed in SH-SY5Y cells treated with 9-*cis* RA, making the induction of cell survival by the activation of NFKB1 improbable in this cell line. However, treatment with ATRA induces expression of BIRC3 in SH-SY5Y cultures but does not modify CARD6 expression levels, thus suggesting the phosphorilation of IKK and NFKB1 and the activation of the survival pathway. The activation of this pathway might be the reason for not detecting a significant increase of apoptosis during the first days of treatment. Besides the activation of the extrinsic pathway, CIDEA overexpression might also contribute to the induction of apoptosis detected after 5 days.

The reduction in the expression levels of TNFRSF1A, TNFRSF25, TP53 and APAF1 is the main result observed after the treatment of IMR-32 cells with ATRA (Figure 7). Since the reduction of APAF1 decreases the levels of the apoptosome and thus the induction of apoptosis by the intrinsic pathway, and TNFRSF1A and TNFRSF25 are receptors that take part in the activity of both the extrinsic and NFKB1 pathways [40], [41], this result suggests the inhibition of those pathways and the existence of a balance among the processes of apoptosis and cell survival. CIDEA is the only proapoptotic gene that we found overexpressed in IMR-32 cells after ATRA treatment. This result, that contrasts with the reduction in its levels observed after the treatment of mature 3T3-L1 adipocites with ATRA [42], [43], is significant as it allows to explain the increase of apoptosis detected from the 7^th^ day on, if we suppose that it is in this moment when the levels of CIDEA are superior to the levels of its inhibitor ICAD.

**Figure 8:**
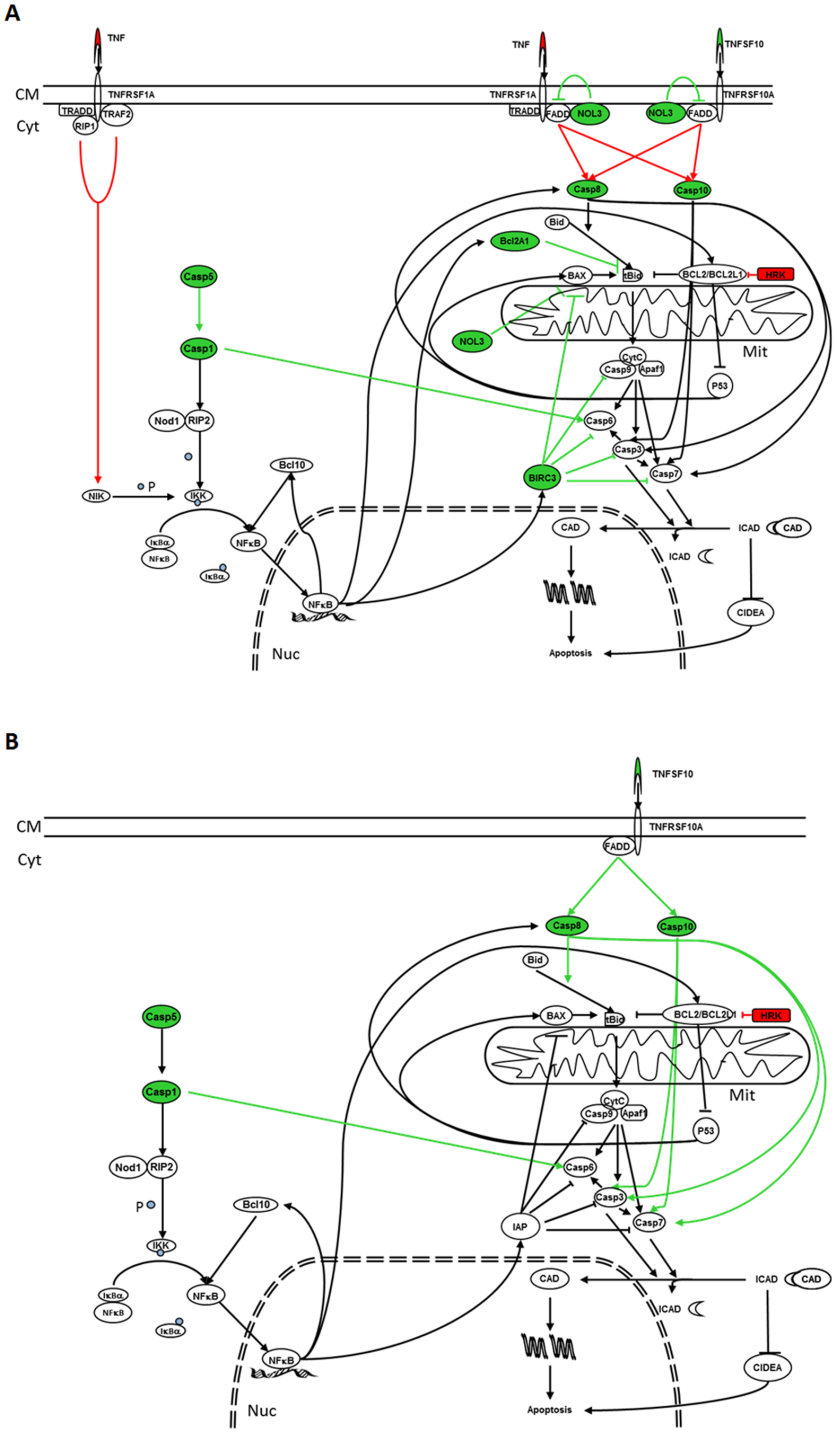
Effect of RA treatment over apoptotic and cell survival pathways in SK-N-DZ RA sensitive cell line. In green colour are represented those genes whose expression has been augmented as a result of 9-*cis* RA (A) or ATRA (B) treatment, while in red are shown those whose expression has been decreased. Green arrows indicate the pathways that will be activated while red arrows indicate the pathways that will be inhibited as a consequence of the modifications of the genes that take part in these pathways. CM: Cell Membrane; Cyt: Cytoplasm; Mit: Mitochondrion; Nuc: Nucleus; P: Phosphorous.

In contrast with the previous observations, analysis of the modifications induced by treatment with RA on the expression profile of genes related to the processes of apoptosis and cell survival in the SK-N-DZ cells suggests that induction of apoptosis by RA in this cell line is a consequence of its action over caspases 1, 5, 8 and 10, whose expression levels were incremented (Figure 8). Moreover, the decrease in the expression level of TNF, and the overexpression of NOL3, an inhibitor of FAS and FADD after 9-*cis* RA treatment, suggest that it is not very likely that the increase of apoptosis is due to the activation of the extrinsic pathway at the level of membrane receptors. Similarly, the overexpressions of NOL3, BCL2A1 and BIRC3, together with the decrease in HRK levels, seem to discard the activation of the intrinsic pathway. Furthermore, the overexpression of caspases 1 and 5 could lead to the phosphorilation of IKK with the subsequent activation of NFKB1, which would explain the decline in the apoptosis induction observed in this cell line after 5 days of treatment.

**Figure 9:**
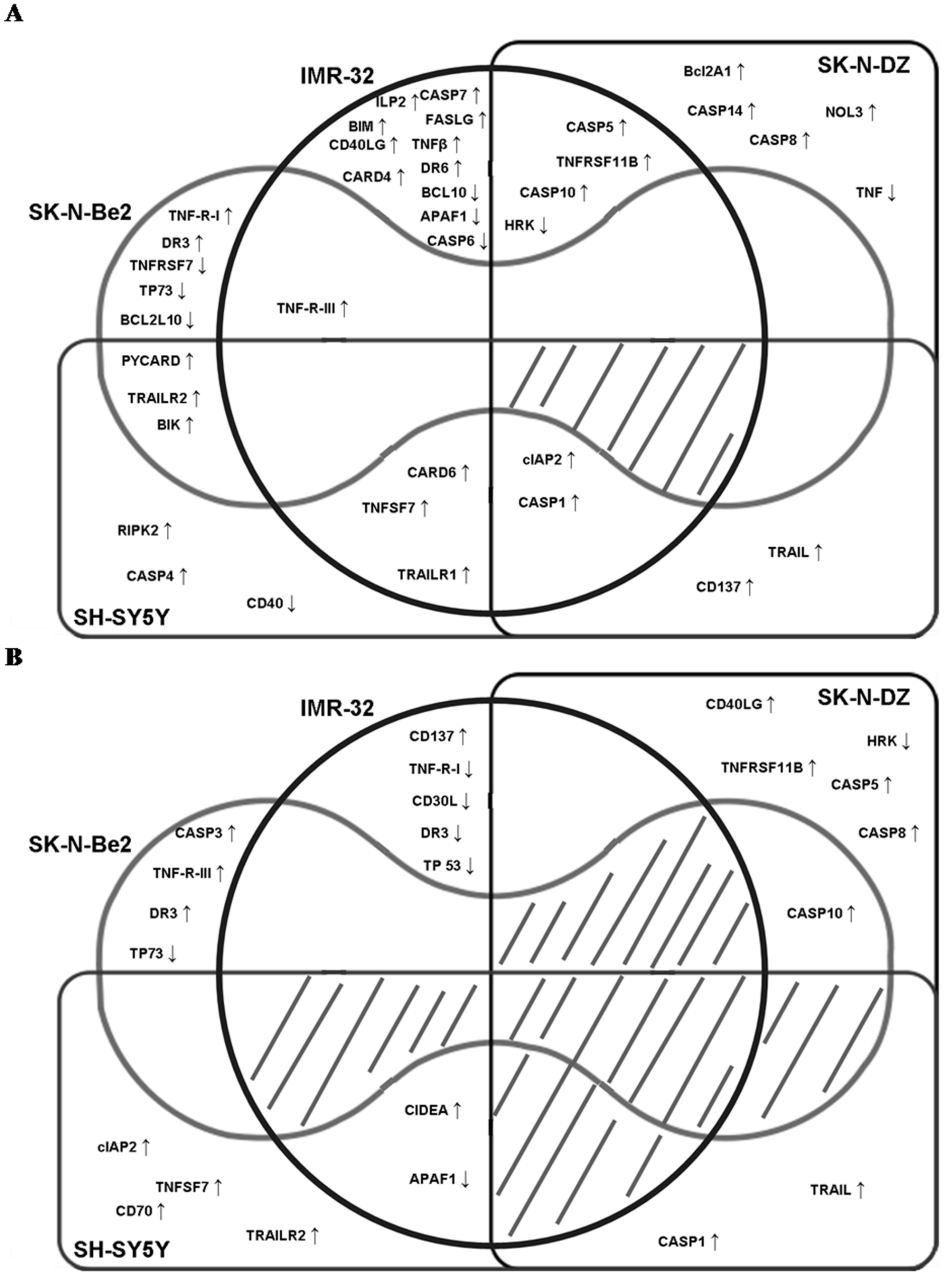
Overlapping pattern of genes modified by RA treatment of neuroblastoma cell lines. Edwards-Venn diagrams showing the genes overexpressed at least 2 times (up arrows) or downregulated by 50% (down arrows) after 5 days of RA treatment of the corresponding cell line. (A) Genes modified by 9*-cis* RA. (B) Genes modified by ATRA.

The overlap among genes whose expression level was modified in a particular cell line after treatment with either of the RA isomers is shown in Figure 4. Only the APAF1 gene was modified by both RA isomers in IMR-32 cells (Figure 4A), while BIRC3, CASP1, TNFRSF10B, TNFSF7 and TNFSF10 were modified in SH-SY5Y cells (Figure 4B), CASP1, CASP5, CASP8, CASP10, HRK, TNFRSF11B and TNFSF10 were modified in SK-N-DZ cells (Figure 4C), and LTBR, TNFRSF25 and TP73 in SK-N-Be(2) cells (Figure 4D). The overlap among genes modified in the different cell lines by each treatment is shown in Figure 9: no gene was modified in every cell line after treatment with either of the isomers. On the other hand, treatment with 9-*cis* RA but not with ATRA induced overexpression of BIRC3 and CASP1 in IMR-32, SH-SY5Y and SK-N-DZ cell lines. Finally, several genes were modified in only one or two of the 4 cell lines after treatments.

Taking together, these results suggest that both 9-*cis* RA and ATRA are able to induce differentiation in neuroblastoma cell lines, while they differ in their capability to induce apoptosis. Furthermore, analysis of the expression profile of apoptosis and survival pathways suggests that the effect of the treatment is cell line specific and depends on the isomer used, being the effect of the 9-*cis* isomer stronger than that of the all-*trans*.

In conclusion, this study contributes to the characterization of the genes of the apoptotic and survival pathways affected as a result of the treatment of neuroblastoma cell cultures with 9-*cis* RA or ATRA. Understanding these mechanisms is important because it would help to understand neuronal development during embryonic stage and the relationship between the cell phenotype and/or the RA isoform in the activation of a determined apoptosis pathway.
